# Improving the quality of life of patients with rheumatoid arthritis after rehabilitation irrespective of the level of disease activity

**DOI:** 10.1007/s00296-020-04711-4

**Published:** 2020-10-01

**Authors:** Daniel Szewczyk, Teresa Sadura-Sieklucka, Beata Sokołowska, Krystyna Księżopolska-Orłowska

**Affiliations:** 1 Departament of Rehabilitation, National Institute of Geriatrics, Rheumatology and Rehabilitation, Warsaw, Poland; 2 Departament of Geriatrics, National Institute of Geriatrics, Rheumatology and Rehabilitation, 1 Spartańska Street, 02-637 Warsaw, Poland; 3 Mossakowski Medical Research Centre Polish Academy of Sciences, Warsaw, Poland; 4 Consultant in National Institute of Geriatrics, Rheumatology and Rehabilitation, Warsaw, Poland

**Keywords:** Disease activity, Quality of life, Rehabilitation, Rheumatoid arthritis

## Abstract

Due to the exacerbation of the disease, the rehabilitation of patients with rheumatoid arthritis is often limited. The aim of the study was to analyze the impact of a comprehensive rehabilitation on a subjective perception of pain and quality of life in patients with rheumatoid arthritis depending on the level of disease activity. The study involved 58 women with rheumatoid arthritis aged 18–60, who underwent a 4-weeks comprehensive rehabilitation program. The assessment included the disease activity level on the DAS28 scale, pain intensity on the 10-point pain scale (VAS) and the value of the CRP protein. The HAQ-DI and KALU questionnaire were used to assess the quality of life. In both groups (group A—DAS28 < 4.2, group B—DAS28 ≥ 4.2) the statistically significant effects in decreasing the level of pain and improvement of quality of life were observed. This indicates the need and effectiveness of rehabilitation regardless of the level of activity of rheumatoid arthritis according to the DAS28 scale. There were no significant changes in the CRP protein level. In conclusion, the rehabilitation of patients with moderate to high disease activity is effective and the success of comprehensive rehabilitation is demonstrated by the decrease of the DAS28 score and the pain level reported by patients, as well as improving self-assessment of health and quality of life.

## Introduction

Rheumatoid arthritis (RA) is a systemic inflammatory disease that leads to functional limitations and premature death [1, 2]. The independence of patients decreases along with the duration of the disease [3, 4]. Most often the onset of RA is symmetrical in small joints. In the early stage of the disease, joints of hands and feet are mostly affected. The inflammatory process develops in synovium leading to destruction in all elements of joint. Patient deals with morning stiffness, pain, joint and periarticular oedema, intra-articular effusion, limited range of motion in joints and as a consequence muscle weakness occurs, which results in the inability to undertake some activities [5–7]. Progression of the disorder and level of disease activity leads to limitations in many activities, also daily life activities, resulting in the increasing disability and loss of self-reliance [8]. The disease affects every aspect of a patient's life. Progressing dysfunctions of the musculoskeletal system significantly worsen a patient's work and social life [9, 10]. Patient and medical personnel must be aware that the patient's condition will be getting worse due to the progressive nature of the disease. Therefore, the main aim of therapy is to reduce the discomfort, improve the quality of life and maintain their independence as long as possible [11]. Pharmacological treatment and an individual rehabilitation program are very important in the treatment process [3, 12]. Both elements are crucial for the best improvement of patient’s health and for slowing down the progression of the disease. Patient’s expectations should take into account the degree of disease advancement and the effects of functional improvement that are real to achieve. The rehabilitation program of a patient with rheumatoid arthritis takes into account the number of affected joints, the degree of their deformation and the level of the destruction of periarticular tissues, as well as limitations in the rehabilitation process [13]. The study attempts to analyze how a comprehensive rehabilitation affects pain and quality of life among patients with rheumatoid arthritis depending on the level of the disease activity.

## Materials and methods

The study involved patients of the Rehabilitation Department of the National Institute of Geriatrics, Rheumatology and Rehabilitation in Warsaw. The study group consisted of 58 women, age 18–60, with diagnosed rheumatoid arthritis according to ACR/EULAR [14, 15] criteria, that underwent a 4 weeks comprehensive rehabilitation program. There were following exclusion criteria: history of surgical procedures within 6 months, alteration in treatment program over the preceding 3 months. The study was approved by the Bioethics Committee at the National Institute of Geriatrics, Rheumatology and Rehabilitation in Warsaw. All patients gave a written consent for participation in the study. The assessment included: disease activity on the DAS28 scale, severity of pain on a 10-point pain scale (VAS) and the value of CRP protein. Quality of life was assessed using the HAQ-DI questionnaire, which consists of questions considering health status in every aspect of daily activities [16] and the KALU questionnaire [17], which assesses the impact of the disease on mobility, social activity, emotional state, daily duties or paid work. In the majority of participants, the disease activity on the DAS28 scale was in the moderate range. In this study, patients were subdivided into two groups depending on disease activity. The threshold, according to which patients were divided for the particular group, was 4.2 in DAS28 (mean value of the moderate activity and mean score in the study group). Group A (moderate disease activity) consisted of 28 patients with DAS28 below 4.2 (min. 1.8; max. 4.19), the mean age in group A was 46 ± 11 years old. Group B (high disease activity) for patients with DAS28 equal or greater than 4.2 (min. 4.24; max 6.62) the group consisted of 30 women and the mean age was 50 ± 8 years old. The disease duration in group A was 13.5 ± 11.2 years (min. 1; max 42), and 18.3 ± 10.5 years in group B (min. 1; max 46). All participants of the study underwent a comprehensive rehabilitation program oriented on the patient’s present problems and needs. Physiotherapy was based on kinesitherapy, which included: load free exercises with or without resistance, individual exercises, whole-body group exercises, exercises in water, hand therapy. Additionally, patients had physical therapy, including electrotherapy, ultrasound, low-frequency magnetic field, laser therapy, and cryotherapy, depending on the patients’ complaints. On average, 5 treatments a day were used in each patient in both groups A and B, while the time and doses were consistent with the applicable methodology of the performed treatments. The exercise time was approx. 30 min for each procedure and patients participated on average in 3 types of exercises each day. The rehabilitation was conducted by 2 experienced therapists. Rehabilitation for each patient lasted 4 weeks (20 treatment days). The evaluation was conducted before and after the program.

### Statistical analysis

Statistical analysis was performed using the STATISTICA 9.0 PL, statistical package software. The values are presented as the mean and standard deviation (SD), and range (min; max). The distribution of variables was non-normal, as stated by the Shapiro–Wilk test. Therefore, to assess the significance of the differences between variables before and after rehabilitation and between A and B groups, the Wilcoxon and Mann–Whitney *U* tests were applied, respectively. The *p* value < 0.05 was considered statistically significant.

## Results

### Disease activity

Analysis of the disease activity parameters on the DAS28 index showed that in group A the disease activity decreased statistically significantly from 3.5 ± 0.6 at the beginning to 3.0 ± 0.8 after treatment (*p* = 0.006). Statistically significant reduction of pain level on the VAS scale was observed, falling from 4.2 ± 2.2 to 2.5 ± 1.8 (*p* < 0.001). In group B the disease activity also decreased statistically significantly from 5.0 ± 0.6 to 4.2 ± 0.8 (*p* < 0.001) as well as the pain level reduced from 5.4 ± 1.6 to 3.7 ± 1.7 (*p* < 0.001). Both groups differed statistically significantly before and after rehabilitation due to these parameters. However, the CRP levels did not change significantly in both groups after the rehabilitation (Table 1).

**Table 1:** Clinical parameters assessed before and after rehabilitation in the study groups

		Group A			Group B		
		Before	After	*p*	Before	After	*p*
DAS28	Mean ± SD (Min; Max)	3.5 ± 0.6 (1.8;4.2)	3.0 ± 0.8 (1.9;4.7)	0.006	5.0 ± 0.6 (4.24;6.63)	4.2 ± 0.8 (2.5;5.9)	< 0.001
		Group A vs B			Before After		< 0.001 < 0.001
VAS	Mean ± SD (Min; Max)	4.2 ± 2.2 (0.7;8.0)	2.5 ± 1.8 (0;7.7)	< 0.001	5.4 ± 1.6 (2.2;8.5)	3.7 ± 1.7 (1;7.5)	< 0.001
		Group A vs B			Before After		0.020 0.008
CRP	Mean ± SD (Min; Max)	8.1 ± 6.3 (1;25)	8.7 ± 8.4 (1;44)	0.883	10.6 ± 9.6 (2;41)	9.9 ± 10.0 (6.5;36)	0.310
		Group A vs B			Before After		0.325 0.975

**Fig.1:**
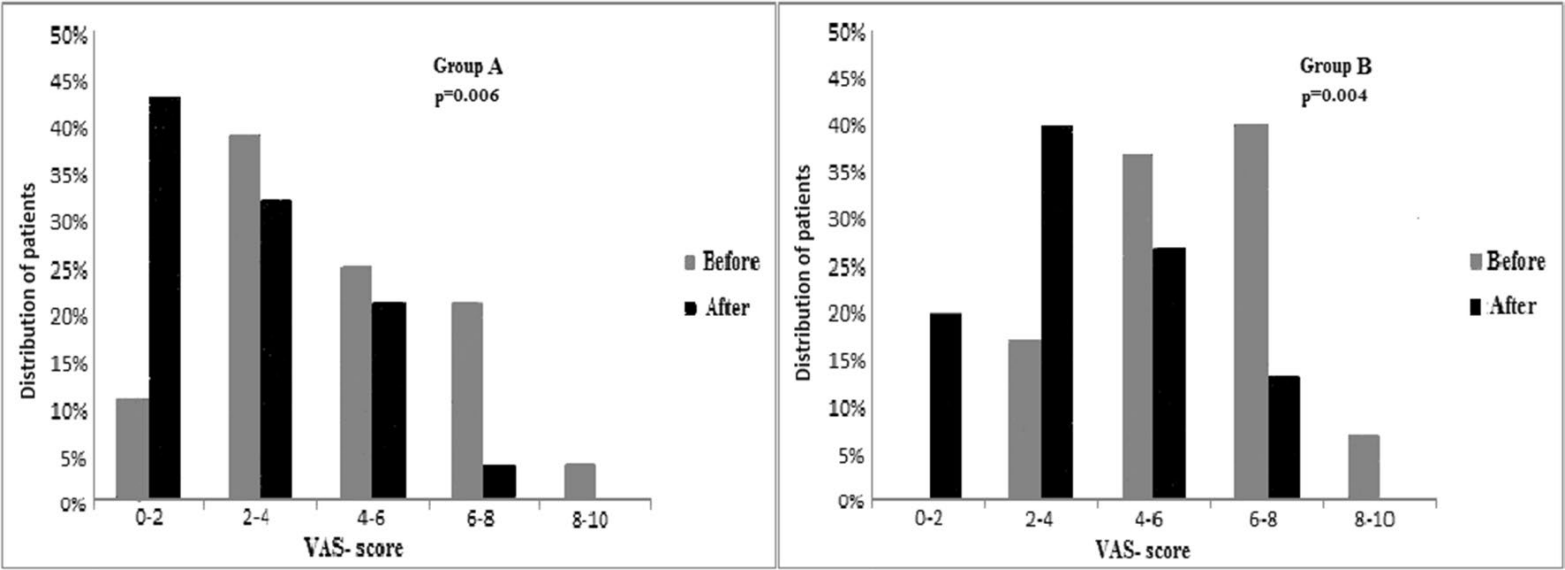
Percentage distribution of patients based on VAS score before and after rehabilitation in the study groups

The average level of pain among the patients in both groups decreased, which results from the shift of the pain level reported by the patients towards the lower values. The distribution of pain level in the individual groups is presented in Fig. 1.

In group A before rehabilitation, a high pain (6–8 VAS) and unbearable pain (8–10 VAS) was reported by 7 patients which is 25% of the study group. Whereas, after the rehabilitation program only 1 patient has reported such intensive pain. Only 3 patients (11%) reported mild pain level (0–2 VAS) before the rehabilitation program, while as many as 12 patients (43%) have reduced the discomfort to no pain or mild pain (0–2 VAS) after the treatment. In group B before rehabilitation, 14 (47%) patients reported high or unbearable pain, whereas after 4 weeks physiotherapy none of the patient-reported unbearable pain (8–10 VAS), high level (6–8 VAS) pain was reported only by 4 patients which is 13%. Before the rehabilitation, there were no patients with mild pain or no pain at all, while after rehabilitation there were 6 such patients (20%).

### Quality of life questionnaires

Based on the results of questionnaires used in this study a significant statistical improvement of the patients’ quality of life and health was observed (Table 2).

**Table 2:** Results of KALU and HAQ- DI before and after rehabilitation in the study groups

		Group A			Group B		
		Before	After	*p*	Before	After	*p*
KALU	Mean ± SD (Min;Max)	0.85 ± 0.47 (0;1.66)	0.57 ± 0.47 (0;1.69)	< 0.001	1.45 ± 1.02 (0.24;6.0)	0.93 ± 0.57 (0.12;2.17)	< 0.001
		Group A vs B			Before After		0.003 0.018
HAQ-DI	Mean ± SD (Max;Min)	0.74 ± 0.67 (0;2.95)	0.47 ± 0.48 (0;1.7)	0.001	1.03 ± 0.52 (0.2;2.2)	0.71 ± 0.53 (0.05;2.15)	< 0.001
		Group A vs B			Before After		0.023 0.040

The KALU questionnaire was used to assess individual functionality. Both groups of patients achieved significantly lower average results after rehabilitation and following a better quality of life. In group A, the average points obtained in the questionnaire decreased from 0.85 ± 0.47 to 0.57 ± 0.47 points, which was statistically significant (*p* < 0.001). In group B, the mean values decreased from 1.45 ± 1.02 to 0.93 ± 0.57, the change was statistically significant (*p* < 0.001). The results obtained in the HAQ-DI questionnaire, which assess disability also showed improvement in the patients’ condition. In group A, the average score decreased from 0.74 ± 0.67 to 0.47 ± 0.48, the change was statistically significant (*p* = 0.001). Also in group B, patients’ outcome was better. The mean values were statistically significantly lower (*p* < 0.001). The score diminished from 1.03 ± 0.52 to 0.71 ± 0.53. Both examined groups differed statistically significantly before and after rehabilitation considering those parameters.

## Discussion

Rehabilitation of patients with RA and the pharmacological treatment are the key elements of successful therapy. Rehabilitation is reducing pain, that is demonstrated in a systematic review done by Park et al. [18], wherein all the analyzed data VAS scale was used for subjective pain assessment, and in all the studies, the statistically significant pain decreased among patients after rehabilitation. Disease activity assessment is a significant element of monitoring the condition of patients with RA. As a disease activity increases, patients’ functional problems increases, self- esteem and quality of life worsen, that was observed in our study based on differences between groups in KALU and HAQ- DI rates. The results showed that regardless of disease activity in DAS28, also in patients’ with exacerbation of the disease, the rehabilitation is needed and remains effective. This is confirmed by the fact that in both assessed groups the average DAS28 index and subjective pain level determined on the VAS scale decreased statistically significantly. Similarly, according to Gizińska et al. [19], after rehabilitation of two groups of patients with high disease activity, statistically significant decreasing of DAS28 level and pain level measured by VAS scale was observed. Our study shows that high disease activity is not a contraindication for rehabilitation due to the fact that those patients get a significant increase in quality of life and life condition. The need and effectiveness of rehabilitation is confirmed in many studies based on the decreasing pain level and disease activity index [20–26]. In the study of Sukharev et al. [27], DAS28 level decreased after rehabilitation in all groups regardless of the rehabilitation program. In our study, also statistically significant changes in those parameters were observed**.** Analysis of the level of CRP after 4 weeks rehabilitation indicated no statistically significant decrease, what is confirmed in a study done by Sadura-Sieklucka [28]. However, Orlova’s [21] research covering a 6-month rehabilitation period shows that there was a statistically significant decrease in the CRP level which may suggest the need to extend the rehabilitation time. Patients’ quality of life level in KALU and health assessment by HAQ-DI showed statistically significant improvement in both questionnaires. KALU questionnaire is an effective tool for assessing patients with rheumatoid arthritis quality of life [17]. In research done by Kowalczyk et al. [17], the mean score in KALU was 1.28 ± 0.55 with the average DAS28 result 5.09 ± 1.05. There was also a significant correlation between the DAS28 index and the KALU questionnaire, which is confirmed by our own research in the group with lower disease activity according to the DAS28 coefficient, a smaller average value of the KALU questionnaire was observed (i.e. DAS28 = 3.5 ± 0.6, KALU = 0.85 ± 0.47, and DAS28 = 5.0 ± 0.6, KALU = 1.45 ± 1.02). Decreasing DAS28 index after rehabilitation reflected also on the decreasing mean value of KALU questionnaire, and that indicates an improvement in the quality of life of patients after rehabilitation. In our study, the mean value of health condition index HAQ-DI also decreased. Rehabilitation significantly influence the improvement of patients health condition and quality of life [29–31]. Research by Ghosh et al. [32] revealed a high correlation between DAS28 level and the mean score of HAQ-DI questionnaire. In research done by Gizińska et al. [19], it was observed that rehabilitation of patients with RA had a positive impact on the reduction of HAQ-DI. Regardless of the rehabilitation method used, the HAQ-DI mean score decreased from 1.82 ± 1.18 to 1.64 ± 1.19 and 2.72 ± 1.48 to 2.12 ± 1.30. This is confirmed by our own study, in both groups regardless the rehabilitation method, the decreasing of HAQ-DI was noticed from 0.74 ± 0.67 to 0.47 ± 0.48 and 1.03 ± 0.52 to 0.71 ± 0.53. The intensity of exercise had no impact on the quality of improvement of patients according to the HAQ-DI index. Studies revealed that exercises focused on improving muscle strength in patients with rheumatoid arthritis had statistically significant impact on achieved results even after 24 weeks [31]. The review of the literature on the subject as well as the present research indicate that the progress in patient rehabilitation is not affected by factors such as disease activity according to the DAS28 indicator or the rehabilitation program [24, 25, 27]. Rehabilitation based on various forms of physiotherapy or training programs gives different results, while all of them prove effective [24]. To sum up, we may conclude that in the rehabilitation of RA patients, the key to success is the right choice of methods and forms of work with a specific patient.

## Conclusions

Rehabilitation in rheumatoid arthritis is a part of treatment that must be carried out from the moment of diagnosis and during periods of exacerbation and remission of the disease. Rehabilitation of patients with moderate to high disease activity remains essential. The effectiveness of a comprehensive rehabilitation is noticable based on the lowering level of DAS28 and lowering pain reported by patients, as well as the improving self-assessment of health and quality of life.
